# Primary angiosarcoma of the ovary with prominent fibrosis of the ovarian stroma. Case report of an 81-year old patient

**DOI:** 10.1186/1746-1596-6-65

**Published:** 2011-07-14

**Authors:** Hans Bösmüller, Christine Gruber, Sophie Haitchi-Petnehazy, Dietmar Wagner, Gerald Webersinke, Steffen Hauptmann

**Affiliations:** 1 Department of Pathology, Krankenhaus Barmherzige Schwestern Linz, Austria; 2 Department of Gynecology, Krankenhaus der Elisabethinen Linz, Austria; 3 laboratory of Molecular Biology und Tumorcytogenetics, I. Internal Department, Krankenhaus Barmherzige Schwestern Linz, Austria; 4 Department of Pathology, University Hospital of Halle-Wittenberg, Germany

## Abstract

Primary angiosarcoma of the ovary (AS) is a rare entity with only 31 reported cases. The majority are pure angiosarcomas, the remainder are associated either with teratomas or conventional epithelial tumors. More than 50% of ovarian AS are disseminated at the time of diagnosis, the minority is detected in stage I. The prognosis of ovarian angiosarcoma in general is poor. Most reports refer to younger individuals, aged from 7 to 46 years, and only 2 case reports could be found for patients older than 64 years. Here we present a very unusual case of angiosarcoma in a 81-year-old patient.

## Background

Primary angiosarcomas (AS) of the ovary are rare malignancies. Approximately 25% of them are associated with other neoplasms, e.g. mature cystic teratoma, [1-3], mucinous cystadenoma, serous and mucinous carcinoma, [4-6], or fibroma [7]. Metastases to the ovary from soft tissue AS rarely occur [8,9]. 60% of all reported AS were detected in stage III and IV [1-4,6,10-18]. Surgical debulking and chemotherapy provided only short disease-free intervals, and most patients died from lung metastasis within 9 months. Twelve reports with stage I disease indicated better outcome, with only two patients dying within one year [3,5,7,17,19-23]. Ovarian AS usually affects premenopausal women or even children [24], with only two reports in postmenopausal individuals [7,18] (Table 1). Here we describe the oldest patient reported with ovarian AS so far.

**Table 1:** Survey of all reported cases of ovarian angiosarcoma including histologic type, stage, author, year of publication and citation number

NR	AGE	HISTOLOGY	STAGE	AUTHOR	YEAR	CITATION
1	7	**AS**	**?**	Evtushenko NT	1958	24
2	19	**AS**	**IV**	Cunningham MJ	1994	10
3	19	**AS**	**III**	Davidson B	2005	16
4	20	**AS**	**I**	Nielsen GP	1997	3
5	21	**AS**	**IV**	Bouchi J	1993	12
6	25	**AS**	**III**	Nucci MR	1998	17
7	25	**AS**	**III**	Lifschitz-Mercer B	1998	22
8	20-32 *	**AS**	**I**	Nielsen GP	1997	3
9	20-32 *	**AS**	**I**	Nielsen GP	1997	3
10	20-32 *	**AS**	**I**	Nielsen GP	1997	3
11	20-32 *	**AS**	**III**	Nielsen GP	1997	3
12	27	**AS**	**I**	Nucci MR	1998	17
13	28	**AS**	**I**	Jha S	2005	20
14	31	**AS**	**I**	Quesenberry CD	2005	21
15	33	**AS**	**IV**	Nara M	1996	15
16	35	**AS**	**IV**	Nucci MR	1998	17
17	38	**AS**	**IV**	Twu NF	1999	11
18	40	**AS**	**IV**	Platt JS	1999	14
19	42	**AS**	**IV**	Patel T	1991	13
20	42	**AS**	**I**	Nucci MR	1998	17
21	46	**AS**	**I**	Furihata M	1998	19
22	67	**AS**	**III**	Bradford L	2009	18
23	81	**AS**	**I**	Bösmüller H	2011	
24	20-32 *	Mature cystic teratoma + **AS**	**III**	Nielsen GP	1997	3
25	20-32 *	Mature cystic teratoma + **AS**	**III**	Nielsen GP	1997	3
26	30	Mature cystic teratoma + **AS**	**III**	den Bakker MA	2006	2
27	32	Mature cystic teratoma + **AS**	**IV**	Contreras AL	2009	1
28	37	Mucinous cystadenocarcinoma + **AS**	**I**	Jylling AM	1999	5
29	45	Borderline serous cystadenocarcinoma + **AS**	**IV**	Pillay K	2001	6
30	77	Mucinous cystadenoma + **AS**	**III**	Ongkasuwan C	1982	4
31	65	Ovarian fibroma + **AS**	**I**	Cambruzzi E	2010	7

* age not specified

## Case Presentation

### Patient

The 81-year-old patient was admitted to the hospital with abdominal pain and distension. Ultra-sonography and computed tomography revealed a huge cystic mass of the right ovary. There were no signs of distant tumor deposits. The patient underwent hysterectomy and adnexectomy on the right, the left adnexa had been taken out decades ago. Further exploration of the abdomen and all other clinical investigations were without pathological findings, six weeks after laparatomy the patient underwent chemotherapy with 4 cycles of doxyrubicin, and after an follow up of 5 months she is still alive, and there are no signs of recurrence.

**Figure 1:**
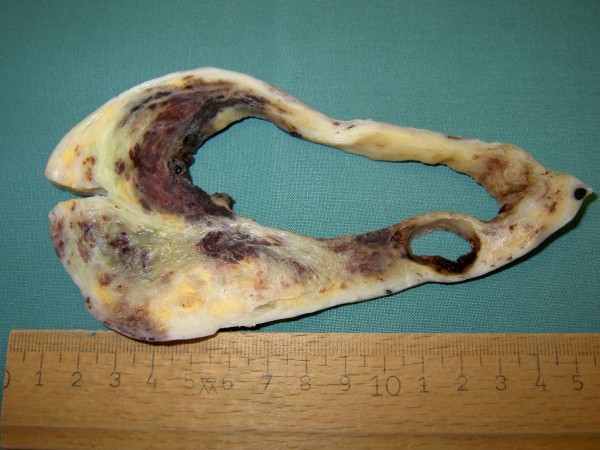
**Cut surface of the right ovary featuring a centrally located tumor associated cyst**.

**Figure 2:**
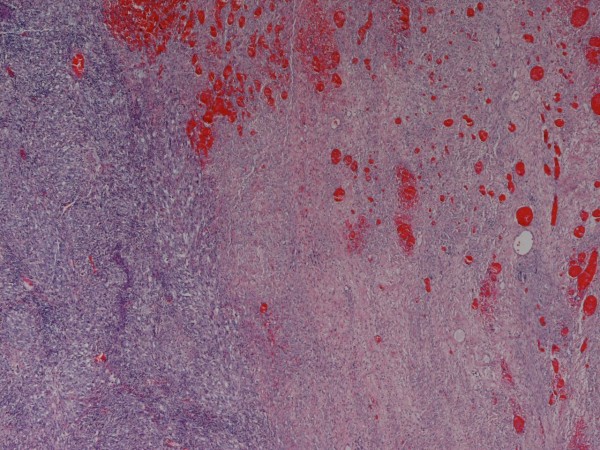
**Transformation from centrally located angiosarcoma high grade (left) to more vasculated and well differentiated tumor areas in the periphery; H&E 40×**.

**Figure 3:**
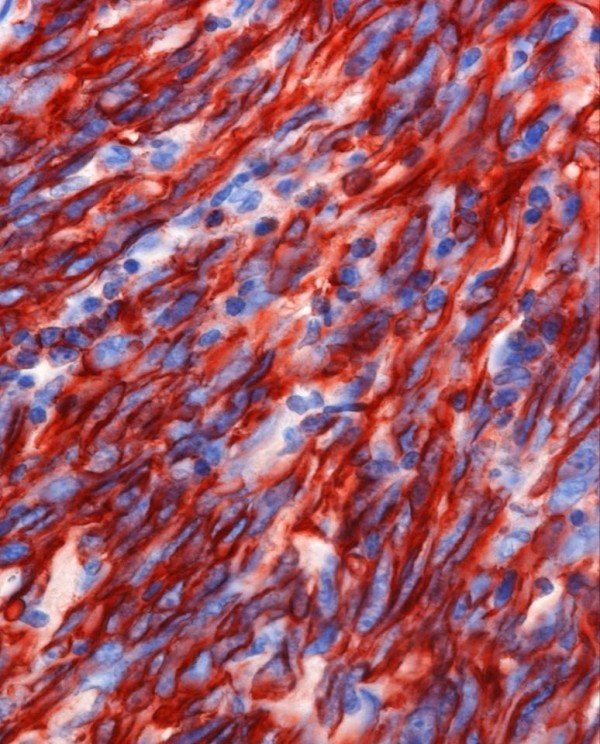
**Centrally located angiosarcoma high grade with fusiform tumor component**. CD31 staining 400×.

**Figure 4:**
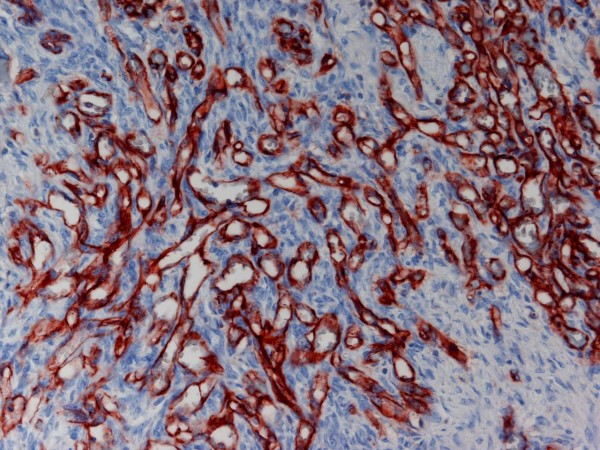
**Atypical vascular proliferation in the cortical areas of the ovary**. CD31 staining 100×.

Macroscopically the tumor had a weight of 2122 grams and measured 30:18:12 cm. The peritoneal surface was inconspicuous. The cut surface showed a big central cyst with a diameter of 14 cm, containing hemorrhagic debris and some luminal projections. The cyst wall and its immediate surrounding consisted of yellowish fibrous tissue with some myxoid glistening changes and hemorrhagic areas, but no significant necrosis (Figure 1). Microscopically, the cyst wall was composed of fascicularly arranged, densely packed atypical spindle cells with pleomorphic nuclei and sparse cytoplasm. Up to 4 mitoses per high power field were counted. Focally, these spindle cells formed Kaposi-like angiomatous spaces containing erythrocytes. Other tumor components had a more epitheloid character. At the periphery a thick fibrose zone was visible with some edema and foci of well formed angiomatous proliferations, lined by atypical endothelial cells (Figure 2, 3, 4). It was interesting to note that the spindle shaped high-grade malignant part of the lesion was restricted to the immediate portion of the tumor surrounding the cyst, whereas the angiomatous proliferation at the periphery was much better differentiated. Intact fibrous ovarian stroma could only be identified in areas bordering the intact peritoneal capsule.

The central highly atypical fusiform tumor infiltrate showed intense staining for CD31, reacted weakly for WT1, but had lost expression of CD34. There were nearly no remaining vascular spaces, and we found a Mib-score of 60%. The more angiomatoid proliferation in the periphery did express both, CD31 and CD34, and Ki-67 was expressed only in some of the atypical endothelial cells (Mib-score < 10%). HHV8, epithelial markers (CK7, CAM 5.2, EMA), and smooth muscle actin were negative. Fluorescent in situ hybridisation for SYT-SSX (X; 18) was performed with LSI SYT Dual Colour Break Apart probe (18q11.2, Abbott, North Chicago, Illinois, USA) and was negative. Based on these findings, the patient was diagnosed with primary angiosarcoma of the ovary, high grade.

## Discussion

**Figure 5:**
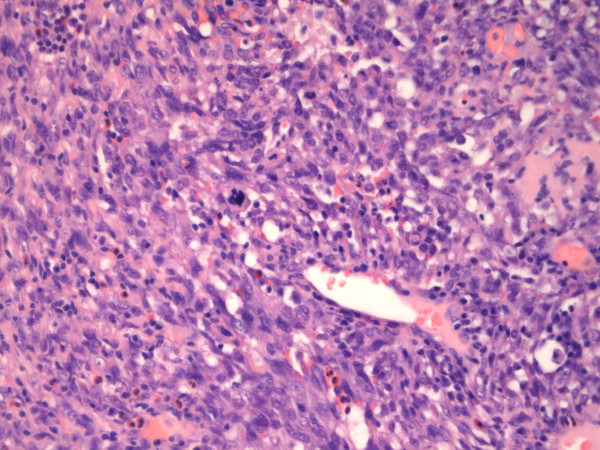
**Centrally located high grade angiosarcoma exhibiting atypical mitosis; H&E 200×**.

Ovarian angiosarcoma (AS) is with rare exceptions a disease of premenopausal woman. Only two patients have been reported in postmenopausal age [7,18] and the 81 years old woman described in this report is the oldest patient with this disease in the literature. AS of the ovary is very rare with only two small case series published so far, one with 4 and the other with 7 cases [3,17]. In both publications ovarian AS were described as morphological heterogenous tumors, a fact emphasized in a few other case reports too. The tumor described in this report represented high grade AS only in its central part, towards the periphery an atypical angiomatous proliferation was obvious, alternating with areas of intense fibrosis. A Mib-score of 60% and the marked pleomorphism with atypical mitotic figures in the central areas are striking features for malignancy, so there was no evidence for reactive angioma (Figure 5). Massive fibrosis may obscure a malignant tumor, leading to the misdiagnosis of fibroma or thecoma, similar to our case in the frozen section diagnosis, but nevertheless AS may coexist with true ovarian fibroma [7]. However, massive hemorrhage usually is present and suggests malignancy. Fusiform and fibrous aspects together with only sparse formation of capillary-like spaces, like in our tumor, may focally mimic myogenous origin or metastasis, respectively, but negativity of actin and expression of vascular markers supported the diagnosis of angiosarcoma. Synovial sarcoma was excluded by negative immunohistochemical staining for epithelial markers and inconspicuous SYT-SSX fluorescent in situ hybridisation [25].

Of 31 reported cases of ovarian angiosarcomas, 23 were pure lesions without coexisting benign or malignant epithelial components. In 5 reports, angiosarcoma was found to be associated with mature cystic teratoma, and in this context it was discussed, whether angiosarcoma is a sarcomatous teratoma, particularly those tumors occurring in younger women[1-3]. In another 3 cases mucinous cystadenoma, mucinous cystadenocarcinoma and borderline serous tumor were coexisting to ovarian AS, rendering the diagnosis adenosarcoma and carcinosarcoma, respectively [4-6], and putting ovarian AS into the context of malignant mesodermal mixed tumor (MMMT) [22]. Angiosarcoma itself may show epitheloid features and can therefore be mistaken for carcinoma or metastasis, and one published case had a predominant reticular growth pattern resembling yolk sac tumor [17].

## Conclusions

These examples show that the suspicion of ovarian AS opens a broad range of differential diagnostic considerations. The correct histopathological diagnosis, however, is of importance because prognosis of ovarian AS is uniformly poor. This could be related to the fact that most patients are diagnosed with advanced disease in stage III or IV. Although patients with stage I disease were shown to survive and even become pregnant [3,20], there are also reports on fatalities of patients with early stage disease.

## Consent

Written informed consent was obtained from the patient for publication of this case report and any accompanying images. A copy of the written consent is available for review by the Editor-in-Chief of this journal.

## Declaration of Competing interests

The authors declare that they have no competing interests.

## Authors' contributions

HB conceived the case report and drafted the manuscript, CG and SP carried out histology and immunohistochemistry, DW supplied clinical data, GW carried out molecular analyses, SH supervised the case report and participated in its design and coordination. All authors have read and approved the final manuscript.
